# The Reduction of Infant Mortality

**Published:** 1907-09-14

**Authors:** J. Theodore Dodd


### EDITOR'S LETTER-BOX.

### THE REDUCTION OF INFANT MORTALITY.

Sir,—The season when infant mortality is the highest has now begun. The causes of the evil are numerous, but undoubtedly one of them is that parents do not call in the doctor soon enough. They ought to obtain a doctor as soon as a child is ill; and, if they are unable to pay for one themselves, they should apply to the relieving officer for an order for the Poor-law doctor. Medical out-relief (unlike ordinary relief) does not disfranchise as regards either the Parliamentary or Municipal vote. The relieving officer may say that he can only give a medical order to persons who are destitute; so it is worth while to quote a letter of the Local Government Board to the Holbeach Guardians* which explains that "where a person suffering from illness is destitute it is the duty of the Guardians, or in the interval between the meetings of the relieving officer, to give such relief as the case may require." The letter then proceeds to say :—

"The test of the Guardians' duty in the matter is the destitution of the patient, and this will not necessarily depend upon his being in the actual receipt of poor relief, but may consist in his being unable to obtain, at his own cost, the requisite medical attendance, nursing, and accommodation."

It will be seen that necessary relief may, and should be, often given to people who are not in the ordinary sense of the word "destitute." I may mention that if the relieving officer refuses to give an order application should be made to the Guardians at their next meeting, or, in a case of "sudden and urgent necessity," to the overseers. It is true that the Poor-law authorities may ward off applications by the "Officer of the House," but few will refuse medical relief to a sick child because his father declines to come into the Workhouse. J. Theodore Dodd.

* The letter will be found in the "Poor-law Officers' Journal," vol. xiv., p. 297, and the "Poor-law Annual," 1907-8, pp. 308-309.

